# Automated 3D segmentation of methyl isocyanate-exposed rat trachea using an ultra-thin, fully fiber optic optical coherence endoscopic probe

**DOI:** 10.1038/s41598-018-26389-2

**Published:** 2018-06-07

**Authors:** Yusi Miao, Joseph C. Jing, Vineet Desai, Sari B. Mahon, Matthew Brenner, Livia A. Veress, Carl W. White, Zhongping Chen

**Affiliations:** 1 Beckman Laser Institute, University of California Irvine, Irvine, 92612 California USA; 2 Department of Biomedical Engineering, University of California Irvine, Irvine, 92697 California USA; 3 Department of Pediatrics, University of Colorado Denver, Denver, 80204 Colorado USA

## Abstract

Development of effective rescue countermeasures for toxic inhalational industrial chemicals, such as methyl isocyanate (MIC), has been an emerging interest. Nonetheless, current methods for studying toxin-induced airway injuries are limited by cost, labor time, or accuracy, and only provide indirect or localized information. Optical Coherence Tomography (OCT) endoscopic probes have previously been used to visualize the 3-D airway structure. However, gathering such information in small animal models, such as rat airways after toxic gas exposure, remains a challenge due to the required probe size necessary for accessing the small, narrow, and partially obstructed tracheas. In this study, we have designed a 0.4 mm miniature endoscopic probe and investigated the structural changes in rat trachea after MIC inhalation. An automated 3D segmentation algorithm was implemented so that anatomical changes, such as tracheal lumen volume and cross-sectional areas, could be quantified. The tracheal region of rats exposed to MIC by inhalation showed significant airway narrowing, especially within the upper trachea, as a result of epithelial detachment and extravascular coagulation within the airway. This imaging and automated reconstruction technique is capable of rapid and minimally-invasive identification of airway obstruction. This method can be applied to large-scale quantitative analysis of *in vivo* animal models.

## Introduction

The development of effective countermeasures to inhalation poisoning by industrial chemicals has been of great interest for several decades. Industrial chemicals, such as methyl isocyanate (MIC), are inexpensive and easy to manufacture, but they can inflict massive damage if released intentionally as an act of terror, or from large-scale accidents or natural disasters, as was the case in the Bhopal disaster^1^. Airways are especially sensitive to MIC, and MIC inhalation can acutely cause airway inflammation and obstruction, pulmonary edema, and death^2,3^. If the victim survives, airway hyperresponsiveness, reactive airway dysfunction syndrome, and/or asthma can occur. Current efforts to develop new therapeutic agents and approaches for MIC rely on animal studies with chemical and structural analysis of MIC-induced damage^4^, *in vitro* testing^3^, histological examination, and arterial blood gas analysis in these models^5,6^. However, anatomical changes in the airway structure during and after exposure to MIC gas have not been directly recorded.

The ability to optimally assess responses to therapeutic interventions necessitates development of efficient, accurate, and quantitative methods for determining airway injury. MRI and CT have been used to visualize airway structure^7,8^. Unfortunately, both MRI and CT tend to be bulky, expensive, and feature insufficient resolution for imaging small airways; thus, they are not optimal for studying airway injuries in small animal models in laboratory settings, and their availability is limited at exposure facilities. Optical coherence tomography (OCT) is a non-invasive imaging technique that uses non-ionizing infrared light to visualize a cross-section of tissue with micrometer scale resolution. Endoscopic OCT probes have been developed to image intraluminal tissues and to get access to the deep structures in the body^9,10^. In the respiratory system, endoscopic OCT has previously been used to quantify airway tissue structure^11–13^, compliance^14^, and lumen caliber^15^. Our group has previously demonstrated anatomical OCT scanning and three-dimensional reconstruction of a human upper airway in patients with obstructive sleep apnea^16^ and quantified respiratory airflow using computational fluid dynamic (CFD) simulation^17^. However, to translate our studies into the airways of rodent models requires a significantly more compact endoscopic probe than previous designs since the diameter of a typical rat trachea is about 6 times smaller than a human’s. Another limitation in previous studies was the manual tracing utilized to reconstruct the 3D structure of the airway from OCT images. This approach was tedious, time consuming, and limited the number and extent of airway analyses that could be performed.

The main challenge for studying airways in a small animal model using OCT is the size of the imaging probe. The maximum acceptable probe diameter is limited by the size of the conducting airways. The average diameter of the rat trachea is 2.8 mm^18^. In addition, animals exposed to MIC are likely to have edema, intraluminal narrowing due to epithelial sloughing and exudate, resulting in airway obstruction, with further compromise of the airway cross-sectional area. Although the traditional 1.5 mm-in-diameter probe with focusing optics provides high resolution and long working distance image, a smaller size is needed for investigating an obstructed airway in small animals in order to preserve the tissue structure. Recently, Lee *et* *al*. proposed an all fiber optic endoscopic design for an ultra-thin imaging OCT probe^19^. This design utilizes a large-core fiber and a stepwise transitional core structure to create a compact and flexible OCT endoscopy probe with a diameter of less than 1 mm. Nevertheless, practical translation of this technology for imaging rat airways exposed to toxic gas remains a challenge because another important aspect of repeatable testing within animal models is an automated measurement and quantitation of airway structure. In order to conduct large-scale animal testing, such as antidote efficacy screening, analysis of OCT images needs to be automated. An automatic segmentation method based on edge detection and graph theory has been recently developed to delineate and quantify the interior airway lining^20,21^. However, those studies have been mostly limited to distinguishing tissue structure, such as thickness in 2D OCT images.

In this report, we optimized the design of a previously developed all fiber optic endoscopic probe specifically for use within rat trachea and applied an automated segmentation algorithm on OCT images to generate accurate 3D reconstructions. In the structural analysis, automatic and quantitative read-outs of trachea volume and cross-sectional area were obtained. The analysis results obtained from automated segmentation were evaluated and compared with manual segmentations. Additionally, for this translational clinical application, we further questioned whether there was any spatial variation in the degree of injury and, if so, whether the obstructed region could be defined within the trachea. This paper details the fabrication process of an all-fiber probe, method of automated segmentation, and analytic results in an MIC-exposed rat model.

## Results

In this study, airways of rats exposed in an MIC inhalation model were first visualized using an all-fiber miniature OCT probe that was specifically optimized for imaging within rat airways. Then, the 3D structure of the intraluminal wall was reconstructed and analyzed from OCT images using an automated segmentation algorithm.

### Fabrication of a fully fiber optic endoscopic probe

**Figure 1:**
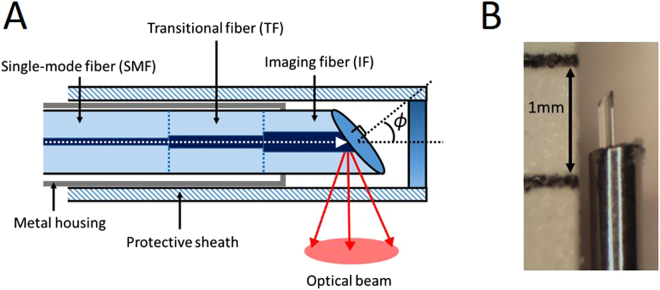
Bare-fiber OCT probe. The probe is made of three optical fibers with core sizes of 9, 12, and 20 um to decrease light divergence and increase imaging range and lateral resolution. The imaging fiber is polished at a 49-degree angle (*ϕ*) to reflect the optical beam. Metal housing is placed around the fiber to reduce the friction during rotation, and a transparent 49tects the probe and the tissue (**A**). The diameter of probe is 0.4 mm and placed in a 24 G protective sheath (not shown) with 0.6 mm outer diameter (**B**).

A flexible, side-scanning, ultra-small endoscopic OCT probe was designed to examine rat tracheas. Since toxic gas inhalation often causes airway obstruction and narrowing, the anatomical structure of the airway is severely restricted, which makes it difficult for most endoscopic OCT probes to pass through. We adopted a previously proposed lens-free probe design^19^ and optimized it for examining the rat airway. In this probe, a stepwise transitional core structure was utilized instead of a focusing lens to reduce the divergence of light and increase the lateral resolution of the probe. This was done by splicing together a series of optical fibers with different core diameters of 9, 12, and 20 um (Fig. 1). The probe did not require any additional focusing optics since the diameter of healthy rat trachea is typically 2.8 mm. In this range, the light divergence is not significant. Additionally, the distal end face of the optical fiber was polished at a 49-degree angle (*ϕ*) to achieve total internal reflection. Based on our previous lens-free probe designs, the polishing angle was slightly deviated from 45 degree to reduce back reflection from the fiber cladding and the sheath surface^19,22^. A metal housing was placed outside the optical fiber to reduce friction during rotational scanning and minimize non-uniform rotational distortion from degrading the image. During scanning, the probe was further protected by a 24-gauge optically transparent sheath so that the endoscopic probe would not damage the airway epithelium during rotation. The outer diameter of the fabricated probe was 0.4 mm, which was much smaller than the previously designed 1.2 mm endoscopic probe used for humans^16^. The probe was proximally connected to an external rotational motor so that it rotated at 1,500 rpm and acquired the 2D images at a rate of 25 frames per second. The probe was pulled back at a constant speed of 5 mm/s along the airway to obtain three-dimensional scanning of the entire trachea. The axial and lateral resolutions of the probe are 6.8 um and 34 um in air, respectively, when the target is placed at a distance of 0.5 mm. Detailed characterizations of the fiber optic probe with a similar design have been described in a previous paper^22^.

### *Ex Vivo* OCT imaging of rat trachea

**Figure 2:**
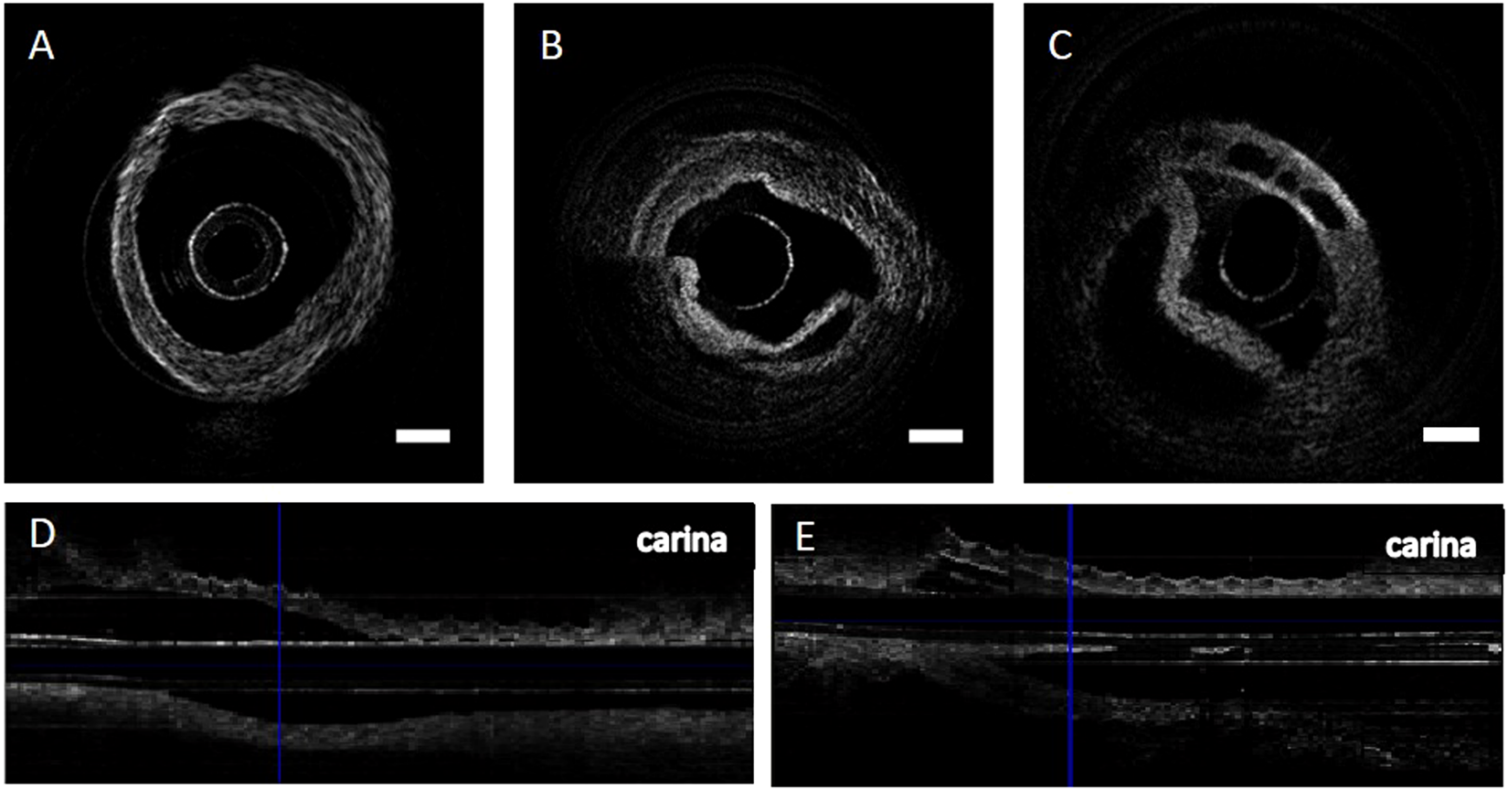
OCT images of rat trachea obtained with a bare-fiber OCT probe. Normal rat trachea has relatively uniform thickness and approximates a circular shape (**A**). MIC-exposed rat trachea has tissue detachment and sloughing (**B**). Another MIC-exposed trachea shows the presence of open spaces within the airway wall (**C**). Longitudinal sectioning of normal trachea shows mostly smooth airway structure (**D**). Longitudinal sectioning of MIC trachea shows obstruction of lumen near the blue vertical line (**E**). Scale bar indicates 0.5 mm.

Using the endoscopic probe described in the previous section, *ex vivo* OCT volumetric scanning of the rat airway was obtained. In order to maintain the original anatomical structure, the rat airway and lung were left intact within the body of the animal after euthanasia. In addition, the rat samples were kept in iced saline-soaked gauze and imaged immediately to prevent structural alteration. Despite the small probe size and simple optics design, our OCT probe captured the entire airway structural image with little attenuation in signal (Fig. 2). The OCT probe was inserted into the anterior nasal airway and carefully guided to the trachea so that the probe would not damage the airway. The distal end of the probe was placed at the carina with scans being obtained continuously up to the nasal cavity at a pullback speed of 5 mm/s. The entire scan took less than 15 seconds to complete.

OCT images of naive rat trachea without MIC exposure were first obtained as a control. As expected, the trachea had a hollow tubular shape with a smooth internal surface (Fig. 2A). Longitudinal scanning also demonstrated a relatively uniform luminal space across the trachea (Fig. 2D). Next, OCT images of trachea from an MIC-exposed rat were analyzed. The cross-sectional images revealed multiple compartments and false lumens within the trachea, which were formed by the partially detached tissue layers (Fig. 2B). In addition, multiple clear spaces were seen within the tracheal wall near the cartilage in some OCT images (Fig. 2C). Longitudinal sectioning of trachea from MIC-exposed rat clearly showed the obstructive tissue near the upper trachea (Fig. 2E).

### Automated segmentation

**Figure 3:**
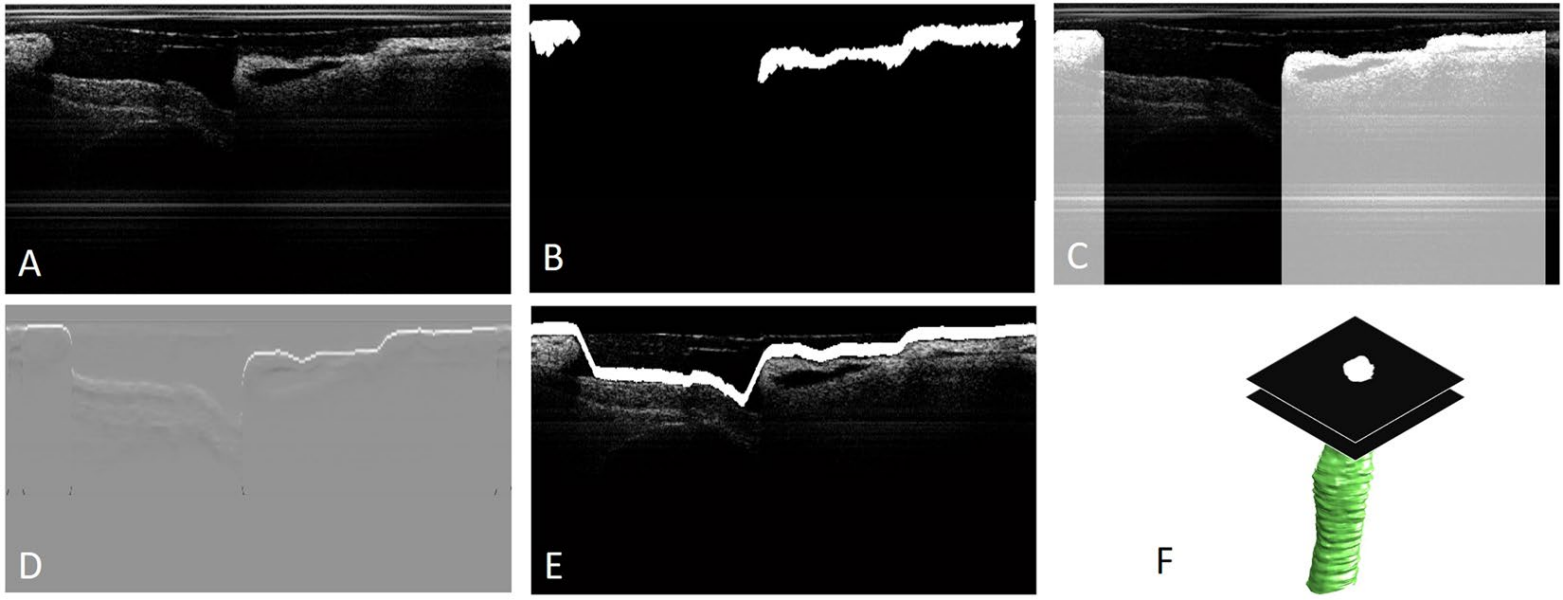
Endoscopic OCT images of MIC-exposed rat airway after de-noising (**A**); binarization reveals the general shape of lumen wall (**B**); emphasizing the most inner lumen surface by creating large pixel intensity differences on the top surface (**C**); first derivative of pixel intensity in the depth detection (**D**); edge detection using dynamic programming (**E**); 3D reconstruction of segmented image (**F**). The inter-frame distance is 0.2 mm.

In order to reconstruct the three-dimensional structure of the airway lumen inner surface, an automated segmentation algorithm was applied to each OCT B-scan image. Afterwards, the 3D surface inner lumen of the airway structure was reconstructed based on the spacing between each frame. The automated segmentation program consists of 3 steps: de-noising, feature extraction, and edge detection. In the de-noising step, noise and artifacts are removed as much as possible to optimize segmentation results. A median filter is applied to suppress speckle noise. In addition, the summation of the pixel intensity in a single A-line is normalized so that the strong reflection from the sheath could be reduced (Fig. 3A). In the feature extraction step, the general structure of the airway is identified, and the most inner (medial) surface is emphasized so that segmentation analysis of the intraluminal surface can be performed. Binarization provides a general shape of the trachea wall (Fig. 3B). In the binary image, small objects are removed so that tissue can be distinguished from artifacts such as the sheath. Additionally, any pixels underneath the tissue are masked and de-emphasized so that only the most inner surface of the luminal wall will be segmented in the following step (Fig. 3C). This prevents the program from detecting another tissue surface caused by luminal detachment. In the last step, the edge of the luminal surface is detected using a dynamic programming (DP) algorithm. The technical details of the dynamic programming have been previously described^20^, and thus, only a brief description is provided in the Methods section. The gradient image is initially obtained by taking the 1st derivative of pixel intensity along the depth (Fig. 3D). Then, the DP algorithm is applied to the gradient image to detect the inner luminal surface (Fig. 3E). Since we apply a mask to emphasize the top layer of the tissue in the OCT image, the DP algorithm only identifies the intraluminal space in which the probe is placed. Once the intraluminal wall is segmented with edge detection, we create a stack of binary images with only the inner luminal space highlighted. Then, the stack of the binary images of inner luminal area is reconstructed into a 3D volume (Fig. 3F).

### 3D model generation and structural analysis

**Figure 4:**
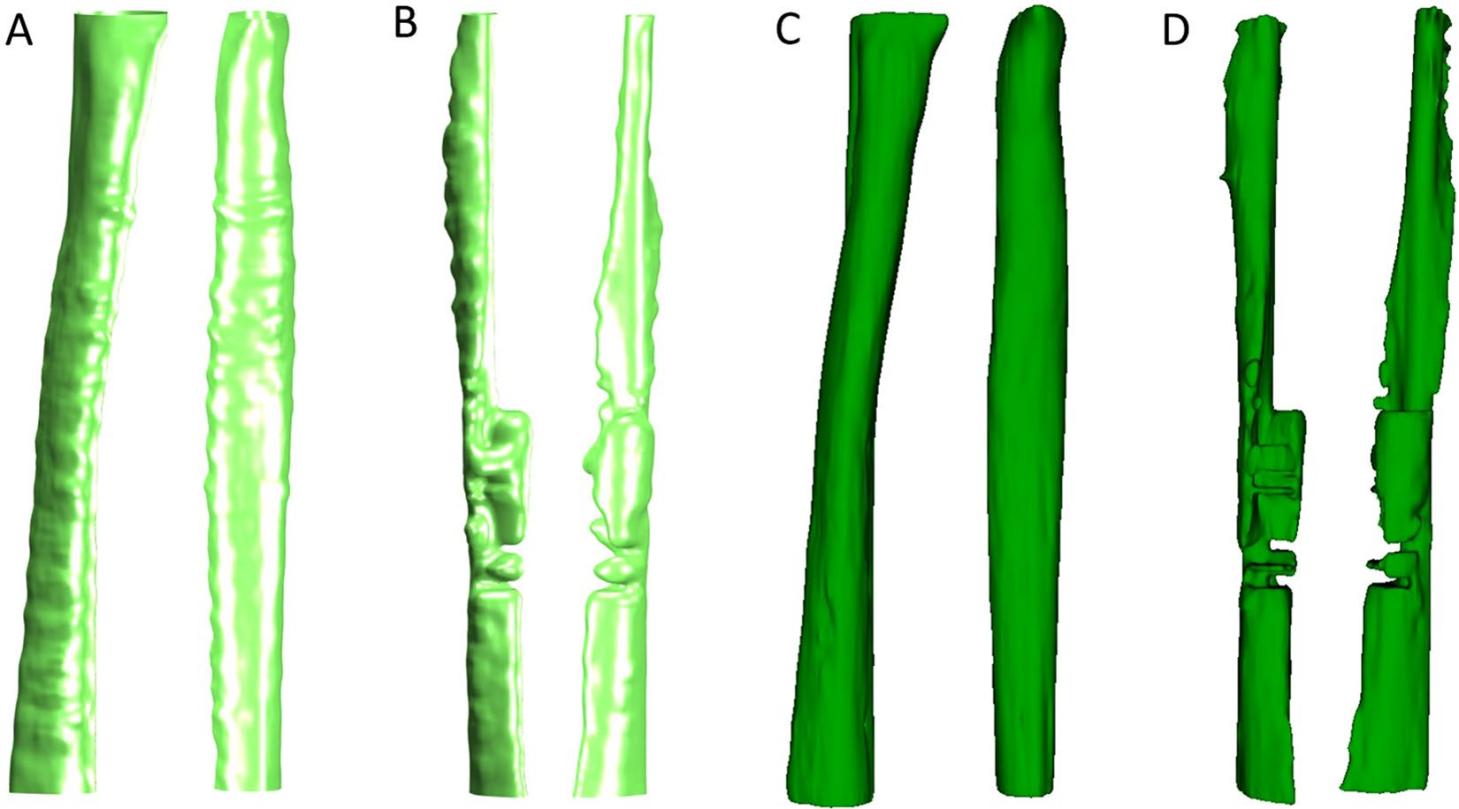
3D reconstruction of rat trachea using automated (**A**-normal, **B**-MIC exposed) and manual segmentation (**C**-normal, **D**-MIC exposed). Normal rat trachea has a relatively uniform diameter with a tubular structure in both the automated (**A**) and manual reconstructions (**C**). The epiglottis opening can be located at the top of the reconstructed trachea (**A**,**C**). MIC-exposed rat trachea shows airway narrowing, especially at the upper trachea (**B**,**D**).

A 3D mesh and surface model of the tracheal structure, from the carina to the level of the epiglottis, was reconstructed from the OCT images (Supplementary Video). A total of 150 B-scan images were segmented to reconstruct a trachea of 3 cm in length for the control and MIC-exposed rat models. In the structural analysis, the volume and cross-sectional area of the intraluminal space across the airway were quantified. The control shows a relatively uniform luminal area across the trachea with an unobstructed air pathway. In contrast, the MIC-exposed rat trachea shows substantial regions of airway narrowing at the level of the epiglottis compared to the control trachea (Fig. 4A,B). The total tracheal volume was estimated to be 115 mm³ and 54 mm³ for control and MIC, respectively. This demonstrated that MIC caused considerable reduction in the airway cross-section area (−2.27 mm²) and volume (−61 mm³) that could lead to airway compromise.

To evaluate the accuracy of the automated 3D segmentation algorithm, the manual segmentation was conducted from OCT images using 3D Slicer (Version 4.6 available from https://www.slicer.org/)^23,24^. Two readers, without prior knowledge of the automated 3D segmentation results, were recruited to manually reconstruct 3D surface models of control and MIC-exposed trachea (Fig. 4C,D). The tracheal volumes obtained from manual segmentation of reader 1 were 109 mm³ and 59 mm³ for control and MIC, respectively. The trachea volumes obtained from manual segmentation of reader 2 were 108 mm³ and 53 mm³ for control and MIC, respectively. While the automated models show a ribbed structure similar to the cartilage rings, the manual models have a smooth surface.

## Discussion and Conclusion

The airway is sensitive to toxic chemicals, and the inhalation of methyl isocyanate (MIC) can cause edema, fluid exudation into airways, airway obstruction, deformity or stenosis, and lead to death^2,3^. Animal models have been developed and evaluated in order to understand the effect of this toxic gas and for development of new therapeutic agents. The toxic-inhaled rat model has especially been commonly utilized to conduct inhalation toxicity studies and investigate variable exposure periods and concentrations^25,26^. Costs can be substantially reduced in the rat model versus other medium- and large-sized animal models, making it easy to screen and compare several potential countermeasures or drug interventions at once. Since MIC directly affects airway lumen tissue and causes obstruction, it is critical to investigate the extent and location of injury and to be able to assess potential aerodynamic changes during and after exposure. Beckett investigated the respiratory effects caused by MIC in Bhopal patients with simple lung function measurements^27^. Stevens *et al*. demonstrated severely compromised lung function and persistent airway obstruction in rats using the nitrogen washout method^28^. However, direct and three-dimensional visualization of the airway structure is required to fully understand the site(s) and aerodynamics of airway obstruction and obtain accurate, quantitative measurements to facilitate development of effective treatments. Tracheal structure of small animals with MIC-induced injury has not been characterized *in situ* mainly due to the size restriction of the probe. In this study, we were able to successfully perform 3D structural analysis of rat trachea after exposure to inhaled MIC gas using a miniature probe design and an automated segmentation algorithm.

A fully fiber optic endoscopic probe was first designed so that the probe could scan rat trachea with MIC inhalation-induced injury. Our endoscopic probe provides high enough spatial resolution to reveal the morphological abnormalities in the tissue structure of rat airway and is small enough to cause no apparent tissue alteration during the probe insertion. Cross-section and longitudinal section of OCT images clearly demonstrated the obstruction and tissue detachment in MIC-exposed rat trachea. The detachment of tissue was not induced by the probe insertion but by the MIC exposure, as we can see from the histological examination in the MIC-exposed rat trachea without probe insertion (Supplementary Fig. S1). In addition, repeated OCT scanning of the same MIC-exposed trachea shows no apparent structural alteration, suggesting that it is not likely itself to cause further obstruction. We imaged the rat tracheas without any fixation to avoid structural and volume changes due to chemical treatment^29^. Prior to imaging, the trachea was kept intact with the rest of the body in cold saline-soaked gauze so that the sample was close to *in vivo*. We have demonstrated this imaging technique is minimally invasive and can be applied to facilitate large-scale *in vivo* testing or longitudinal evaluation of airway obstruction.

**Figure 5:**
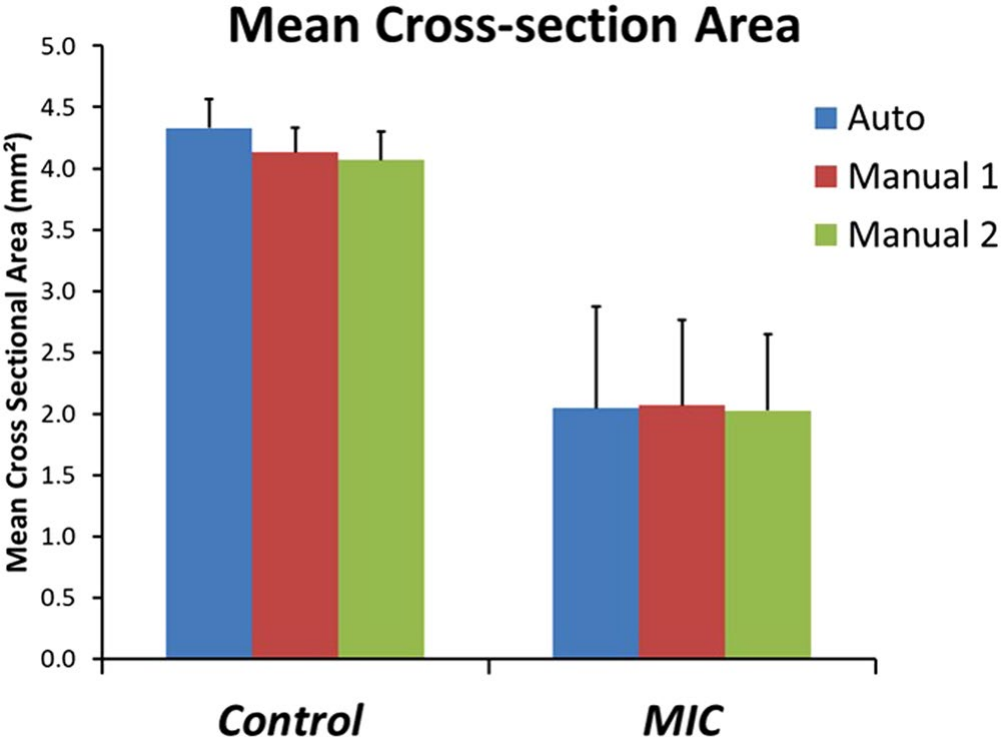
Cross-sectional area of the trachea. Automated segmentation shows 4.33 ± 0.23 mm^2^ for control and 2.05 ± 0.82 mm^2^ for MIC inhalation. Manual segmentation of reader1 resulted in 4.13 ± 0.20 mm^2^ for control and 2.07 ± 0.69 mm^2^ for MIC inhalation. Manual segmentation of reader2 resulted in 4.07 ± 0.23 mm^2^ for control and 2.03 ± 0.61 mm^2^ for MIC inhalation. Error bars show standard deviation. MIC samples have a larger standard deviation due to the non-uniform airway cross-sections and obstruction of the airway.

**Figure 6:**
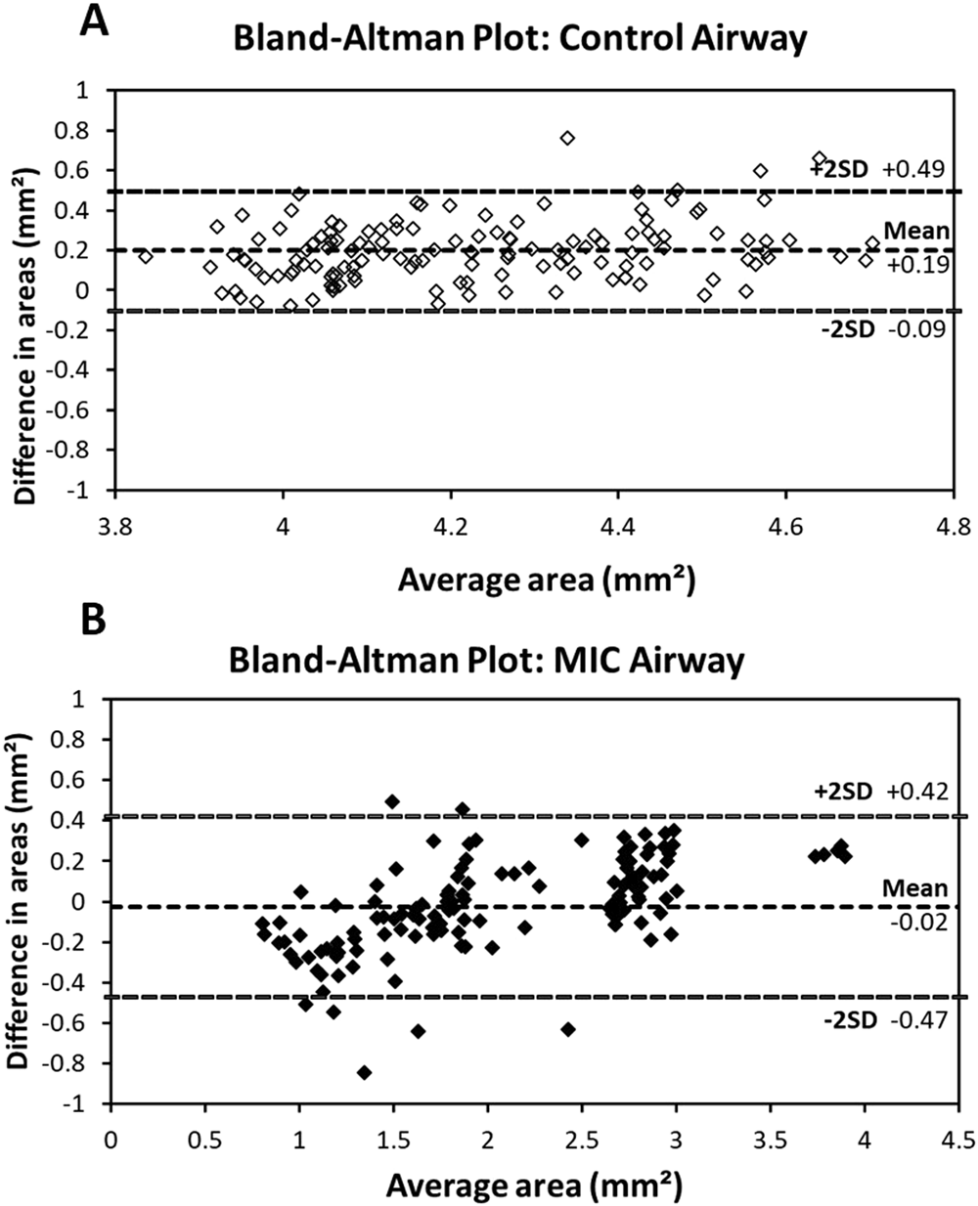
Bland-Altman plots show the differences in the cross-section areas between automated and manually segmented airways from OCT images in the y-axis, and the average areas of the two in the x-axis. Both control (**A**) and MIC airways (**B**) show that there is very little bias (<5%), indicating good correlation between manual and automated segmentation results; 95% limits of agreement and bias versus mean are shown by dashed lines.

In order to assess the accuracy and robustness of the automated algorithm, the 3D-reconstructed airways from the automated segmentation were compared to the manual segmentation results. The manual tracing provided similar results to the automated algorithm in terms of mean cross-sectional area and standard deviation for both the control and the MIC-exposed rat (Fig. 5). Smaller mean cross-sectional and large standard deviation in the MIC sample resulted from narrowing and variability of injury within the airway due to the effects of the toxic gas exposure. As there is no “gold standard” for comparison to the automated segmentation method, Bland-Altman analysis (Fig. 6) demonstrated close correlation without a systematic relationship between the difference in luminal size obtained via automated versus manual results based on location or diameter. The Bland-Altman plot of the control also indicated that the manual segmentation results are consistently slightly smaller than the automated across the frames, which is due to the human eyes tracing the most obvious lines and therefore lacking details in the manual tracing. This is also evident from the absence in tracheal rings in the manually generated 3D airway model. Another contributing factor to the differences in the manual versus automated results is that the automated segmentation in the MIC sample provides conservative estimates of the luminal area in the “noisy” region, and it occasionally estimates the cross-sectional area to be smaller than the actual area due to airway edema and luminal debris that are scattered within the trachea. The automated segmentation of upper trachea in the MIC sample, where the airway is severely obstructed and “noisy,” indicated a lower mean cross-sectional area than the manual results despite that manual tracing tends to give smaller estimates in the normal regions. This may be desirable in some cases since the tissue debris and fluid accumulation substantially compromise the airway function and cause airflow obstruction. However, the automated segmentation algorithm can be calibrated as well so that it covers a larger region and provides more accurate assessment of only the intraluminal cross-section areas. Additionally, artificial neural networks can be implemented to better differentiate the difference between the lumen wall and the fluid and/or debris that are situated inside the airway. Furthermore, in some cases, additional “lumens” or “false lumens” are created by separation and/or sloughing of tracheal tissue layers. The degree to which these lumens conduct airflow is likely to be variable. The automation algorithms can also be modified to map the secondary lumens if clinically useful. We propose that automated segmentation may outperform manual tracing, especially when handling large data sets, since it will not be subject to intra- and inter-reader variation, and will significantly decrease the processing time.

The 3D reconstruction results from MIC-exposed rat trachea revealed differences in the degree of obstruction between the upper trachea and lower trachea. The upper trachea in the MIC-exposed animal showed significant narrowing compared to the normal trachea while the lower trachea only showed moderate airway narrowing (Supplementary Fig. S2). It is possible that the upper trachea and lower trachea near bronchi react to the chemicals differently, and exposure to MIC, a highly reactive and readily absorbed molecule, would be expected to impact more proximal airways to a greater extent^30^. It could also be due to other differences in the deposition or sensitivity of airway regions to specific chemicals. Our previous study showed that there are differences in the degree of smoke inhalation-induced airway injury between upper and lower trachea in a rabbit model^31^. However, large-scale animal studies are needed to substantiate the biological mechanisms of MIC injury, and that is beyond the scope of this paper.

In this study, we have evaluated a fully fiber optic miniature OCT probe for scanning the airway in small animal models and developed an automated 3D segmentation algorithm for reconstructing a 3D airway structure with minimum human intervention. We demonstrated that *ex vivo* rat trachea with MIC inhalation induced injury can be analyzed in 3D, and physiological parameters, such as tracheal volume and the cross-sectional area, can be obtained. If performed immediately upon procurement of the sample, the technique also could be used to quantitate airway obstruction without lung fixation. We are now prepared to use this approach for screening chemical countermeasure effectiveness that will have more focus on the biological mechanisms of MIC injury. Furthermore, this approach should provide enabling technology for additional study directions. For example, functional information could then be obtained from computational simulation such as computational fluid dynamics analysis. Since the probe is less than 0.5 mm in diameter, this technique should be applicable to investigating small animal models *in vivo* without necessitating sacrifice of the animals. Investigating the animals serially at different time points without sacrificing has many advantages since the toxic substances usually affect the airway in a time-dependent manner, and recovery may be slow or delayed, and late phase complications can occur. However, airway motion due to breathing as well as cardiovascular pulsation could both cause motion artifacts. In addition, animals in this study, following inhalation of high levels of MIC, have substantial respiratory distress and hypoxemia. Introducing even a small endoscopic probe can cause profound obstruction, severe distress, and even greater motion artifacts. Therefore, additional effort, practice, and, possibly, an even thinner probe are required to complete the study such that the rat can be re-awakened and studied again subsequently. This imaging technique could also be combined with other techniques used in OCT to open up areas for functional information investigations of the trachea in small animal models. OCT could potentially be combined with fluorescence imaging to investigate depth of injury, epithelial regeneration and healing, and inflammatory response^32,33^. Compliance and viscoelasticity of airway tissue also could be investigated with optical coherence electrography. Different light sources can be utlized to invest rat airway with higher resolution^34^ or deeper penetration depth^35^. In addition, ultrasound could be used for displacement in the tissue from external sites to investigate its material properties^36,37^.

## Methods

### MIC synthesis and rat inhalation exposure model

Methyl isocyanate (MIC) was prepared on site at MRIGlobal (Kansas City, MO) using the Curtius Rearrangement method by refluxing acetyl chloride with sodium azide in toluene until the starting materials were consumed. It was demonstrated to be 99.2% pure by gas chromatography-flame ionization detection (GC-FID). For exposure of rats, the entire system was contained within a fume hood. Male Sprague-Dawley rats (250–300 g) were exposed in a nose-only (CH Technologies) system. MIC vapor was generated using a custom vapor diffusion proprietary system (MRIGlobal) and delivered to the plenum of the exposure system in a mixture of dry nitrogen (50 mL/min UHP nitrogen) that was subsequently blended with carbon- and HEPA-filtered dry air (10–15 L/min depending on desired dose). Downstream from the site of mixing, gas constituents were monitored via access of a 3-way valve by Fourier transform infrared spectroscopy (FTIR) and subsequently delivered to the plenum of the CH Technologies system. Exhaust from that system was subsequently scrubbed in 10% sodium hydroxide and exhausted through the hood via a pump. All procedures were performed under the approval and in accordance with the regulations of Institutional Animal Care and Use Committee (IACUC) at MRIGlobal (Kansas City, MO) under protocol 112515(06)1E.

### Optical coherence tomography imaging system

Our imaging system is based on Fourier domain OCT and utilizes a 50 kHz swept-source laser (1310 nm, AXSUN Technologies, Billerica, MA) with a bandwidth of 110 nm. The output beam from the laser is first split by a 90/10 coupler into the OCT sample arm and the reference arm. The reference arm has an optical delay line and a Faraday mirror to adjust the optical path length and create interference with the light in the sample arm. The sample arm consists of a fiber optic rotary joint, a motorized linear pullback stage, and an endoscopic probe to achieve volumetric scanning of the rat airway. Dual circulators direct the reflected light from the sample and reference arm to a 50/50 coupler, and the OCT interference fringe is detected by a 1.6 GHz wide balanced detector. Finally, the signal from the detector is sampled using a 12 bit data acquisition card (500 MHz, Alazar Technologies Inc., Pointe-Clare, Quebec, Canada). The emitting power of the probe was 5.8 mW, and the system sensitivity was estimated to be 102 dB at the time of the measurement.

### Dynamic programming (DP) algorithm

The technical details of the dynamic programming have been previously described^20^, and thus, only a brief description is provided. In the DP algorithm, cost values are assigned to each pixel of the input image as below.

1 $$Cost(i,j)=min[\begin{array}{c}\alpha \cdot w(I(i-1,j+1),I(i,j))+Cost(i-1,j+1)\\ w(I(i-1,j),I(i,j))+\,Cost(i-1,j)\\ \alpha \cdot w(I(i-1,j-1),I(i,j))+\,Cost(i-1,j-1)\end{array}]$$

where $\alpha$ is a scale factor, $I(i,j)$ is an intensity value at the pixel location $(i,j)$, and

2 $$w(a,b)=2\cdot \,\max (I)-a-b.$$

The cost values are estimated from the left corner. Once all the costs values are assigned, the edge can be found by following the pixels with minimum costs starting from the right-most column. Using 8-neighbor connectivity, the path with the minimum cost is the edge in the graph. In order to maintain the connectivity of the path at the both ends, the OCT image is duplicated and concatenated at the both ends before applying the algorithm.

## Electronic supplementary material

Supplementary Video

Supplementary Information
