# Supplementary material for: Automated 3D segmentation of methyl isocyanate-exposed rat trachea using an ultra-thin, fully fiber optic optical coherence endoscopic probe
Source: Sci Rep. 2018 Jun 7;8:8713. doi: 10.1038/s41598-018-26389-2 (PMC5992171; doi:10.1038/s41598-018-26389-2)
Supplement: Supplementary file 2 — Supplementary Information [file 41598_2018_26389_MOESM2_ESM.pdf]

Automated 3D segmentation of methyl isocyanate-exposed rat trachea using an ultra-thin, fully fiber optic optical coherence endoscopic probe

Yusi Miao[1,2], Joseph C. Jing[1,2], Vineet Desai[1], Sari B. Mahon[1], Matthew Brenner[1], Livia A. Veress[3], Carl W. White[3] & Zhongping Chen[1,2*]

1 Beckman Laser Institute, University of California, Irvine, Irvine, California 92612
2 Department of Biomedical Engineering, University of California, Irvine, Irvine, California 92697
3 Department of Pediatrics, University of Colorado Denver, Denver, Colorado 80204

*Corresponding author:

Zhongping Chen, PhD
Professor
Dept. of Biomedical Engineering
University of California, Irvine
Irvine, CA 92617
e-mail: z2chen@uci.edu

Supplementary Figures

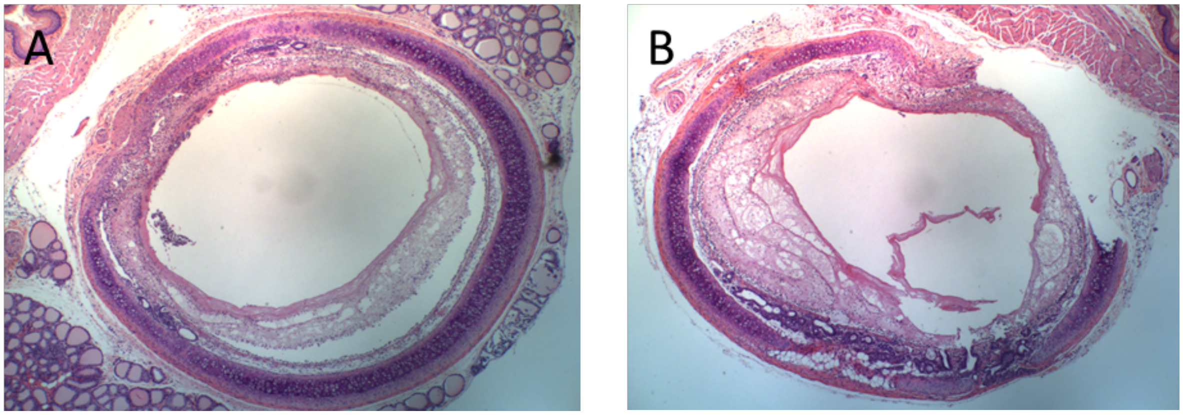

Supplementary Figure S1. Histological sectioning of rat trachea exposed to MIC gas shows the detachment of lumen from cartilage (**A**) and erosion of cartilage and stenosis (**B**).

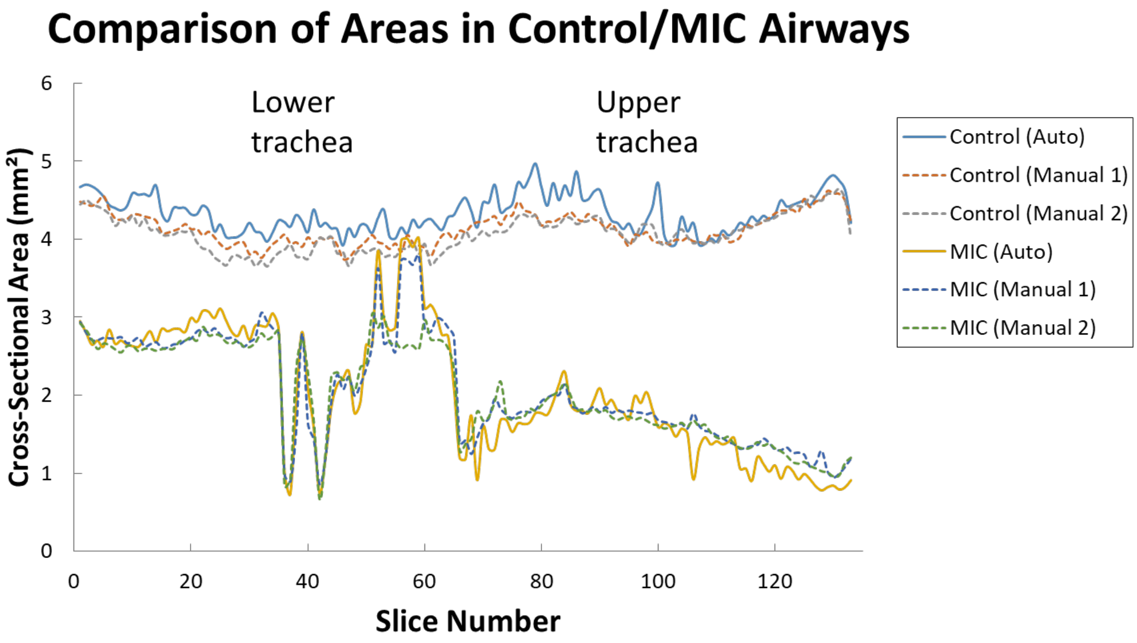

Supplementary Figure S2. The plot of cross-section area from carina (slice 0) to epiglottis opening (slice 130). While the control shows consistent cross-section area, the MIC-exposed trachea shows significant narrowing near epiglottis. The OCT slices are 0.2 mm apart.

Supplementary Video Legend

Supplementary video: 3D surface models of rat trachea obtained from automated segmentation of OCT images.
